# Further results involving Marshall–Olkin log-logistic distribution: reliability analysis, estimation of the parameter, and applications

**DOI:** 10.1186/s40064-016-2007-x

**Published:** 2016-03-31

**Authors:** Arwa M. Alshangiti, M. Kayid, B. Alarfaj

**Affiliations:** Department of Statistics and Operations Research, College of Science, King Saud University, P.O. Box 2455, Riyadh, 11451 Kingdom of Saudi Arabia

**Keywords:** Marshall–Olkin, Reversed hazard rate function, Mean residual life function, Mean inactivity time function, Maximum likelihood estimates, Applications

## Abstract

The purpose of this paper is to provide further study of the Marshall–Olkin log-logistic model that was first described by Gui (Appl Math Sci 7:3947–3961, 2013). This model is both useful and practical in areas such as reliability and life testing. Some statistical and reliability properties of this model are presented including moments, reversed hazard rate and mean residual life functions, among others. Maximum likelihood estimation of the parameters of the model is discussed. Finally, a real data set is analyzed and it is observed that the presented model provides a better fit than the log-logistic model.

**Electronic supplementary material:**

The online version of this article (doi:10.1186/s40064-016-2007-x) contains supplementary material, which is available to authorized users.

## Background

Many researchers strive to introduce new families of distributions or to generalize existing distributions, which can be used to describe the lifetimes of devices or to describe sets of real data. In Marshall and Olkin (1997), Marshall and Olkin (M–O) introduced a new family of distribution in an attempt to add a parameter to a family of distributions. Let *X* be a random variable with survival function ${\overline{F}}(x)=1-F(x)$, then

1 $$\begin{aligned} {\overline{G}}(x;\alpha )&= {} \frac{\alpha {\overline{F}}\left( x\right) }{1-{\overline{\alpha }}{\overline{F}}(x)} \nonumber \\&= {} \frac{\alpha {\overline{F}}(x)}{F(x)+\alpha {\overline{F}}( x)},\quad -\infty \le x\le \infty ,\quad \alpha >0, \end{aligned}$$

is a proper survival function called M–O family of distributions where ${\overline{\alpha }}=1-\alpha$. Clearly, Eq. (1) provides a tool to obtain a parametric distribution from existing one. The probability density function (pdf) of the general M–O family, say *g*(*x*), takes the form

2 $$g(x;\alpha )=\frac{\alpha f(x) }{\left[ 1-{\overline{\alpha }}{\overline{F}}(x)\right] ^{2}},\quad -\infty \le x\le \infty ,$$

where *f*(*x*) is the pdf corresponding to *F*(*x*). Applications, properties and applications of M–O extended distributions can be found in Alshangiti et al. (2014, 2016), Okasha and Kayid (2016), Ghitany et al. (2007), Ristic et al. (2007), El-Bassiouny and Abdo (2009, 2010), Srinivasa et al. (2011), Jose and Krishna (2011), Lin and Li (2012), Cordeiro and Lemonte (2012).

Recently, Gui (2013) introduced and studied the M–O log logistic distribution, denoted by M–O log-logistic. The paper’s objectives are to investigate some statistical and reliability properties of M–O log-logistic distribution and to illustrate its applicability in different areas. The paper is organized into five sections. The density and the moment of the model are given in “Extended log-logistic distribution” section. In that section, we provide some new statistical and reliability functions (reversed hazard rate, mean residual life, mean inactivity time, etc.) and discuss their properties. Furthermore, maximum likelihood estimation problems are considered in “Maximum likelihood estimators” section. To indicate the adequacy of the model, some applications using a numerical example and an example with real data are discussed in “Fitting reliability data” section. Finally, in “Conclusion” section, we provide a brief conclusion and some remarks regarding the current and future research (Additional file 1).

## Extended log-logistic distribution

In probability and statistics, the log-logistic distribution (LLD) (known as the Fisk distribution in economics) is a continuous probability distribution for a non-negative random variable. It is used in survival analysis as a parametric model for events whose rate increases initially and decreases later, for example mortality rate from cancer following diagnosis or treatment. It has also been used in hydrology to model stream flow and precipitation, and in economics as a simple model of the distribution of wealth or income. The LLD is obtained by applying the logarithmic transformation to the logistic distribution in much the same way as the log-normal distribution is obtained from normal distribution or the log-Pearson distribution from the Pearson distribution. The LLD is a special case of Burr’s type-XII and also a special case of the Kappa distribution, that have been applied to precipitation (c.f. Burr 1942; Mielke and Johnson 1973). The survival function of the log-logistic distribution $(\beta ,\gamma )$ takes the form

3 $${\overline{F}}(x;\beta ,\gamma )=\frac{1}{1+\left( \frac{x}{\gamma }\right) ^{\beta }},\quad x\ge 0,$$

where $\gamma >0$, $\beta >1$. Here $\beta$ is a shape parameter and $\gamma$ is a scale parameter. According to Gui (2013), substituting (3) in (1) we get the M–O log logistic distribution, denoted by M–O log-logistic $(\alpha ,\beta ,\gamma )$ with survival function

4 $${\overline{G}}(x;\alpha ,\beta ,\gamma ) =\frac{\alpha }{\alpha +\left( \frac{x}{\gamma }\right) ^{\beta }},\quad 0\le x\le \infty ,\quad \alpha ,\gamma >0,\quad \beta >1.$$

The corresponding CDF and pdf are obtained respectively as

5 $$G(x;\alpha ,\beta ,\gamma ) =\frac{\left( \frac{x}{\gamma } \right) ^{\beta }}{\alpha +\left( \frac{x}{\gamma }\right) ^{\beta }} ,\quad 0\le x\le \infty ,\quad \alpha ,\gamma >0,\quad \beta >1,$$

and

6 $$g(x;\alpha ,\beta ,\gamma )=\frac{\alpha \frac{\beta }{\gamma }\left( \frac{x }{\gamma }\right) ^{\beta -1}}{\left[ \alpha +\left( \frac{x}{\gamma } \right) ^{\beta }\right] ^{2}},\quad 0\le x\le \infty ,\quad \alpha ,\gamma >0,\beta >1.$$

### Statistical and reliability properties

In this subsection, we investigate some statistical and reliability properties of the M–O log-logistic. Let $X\ge 0$ be a random variable representing life with cdf *G* and rf ${\overline{G}}=1-G$ and assume that *G* admits the probability density *g*. The reversed hazard rate (RHR) of *X* is defined by

$$r_{F}(x)=\frac{g(x)}{G(x)},\quad x>0.$$

The RHR function is well-known and useful tool in reliability theory and in other areas of applied probability and statistics. In addition, the RHR function has been receiving increasing attention in the recent literature of reliability analysis and stochastic modeling. The RHR of a random variable *X* with M–O log-logistic $(\alpha ,\beta ,\gamma )$ respectively is

7 $$r(x;\alpha ,\sigma ,\beta ,\gamma )=\frac{\alpha \frac{\beta }{\gamma }}{ \frac{x}{\gamma }\left[ \left( \frac{x}{\gamma }\right) ^{\beta }+\alpha \right] },\quad 0\le x\le \infty .$$

The next result provide the behavior of the RHR of the M–O log-logistic $(\alpha ,\beta ,\gamma )$ distribution, and can be verified using elementary calculus.

#### **Lemma 1**

*Let*$X \sim$*M–O log-logistic*$(\alpha ,\beta ,\gamma )$, *then the reversed hazard rate is decreasing if*$\beta >-1$, *independent of*$\alpha$*and*$\gamma$.

**Fig. 1:**
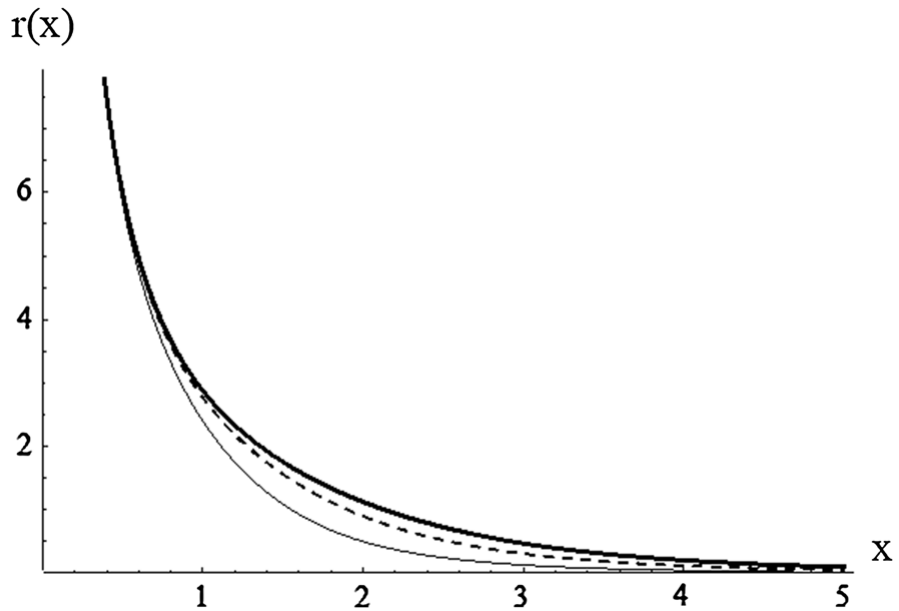
Plot of RHR for ${\alpha =0.5},{1.5,3}$ (*plain*, *dashed*, *bold*), ${\beta =3}, {\gamma =2}$

Figure 1 illustrates some of the possible shapes of the reversed hazard rate functions of M–O log-logistic $(\alpha ,\beta ,\gamma )$ distribution for different values of the parameter ${\alpha }$.

The conditional reliability function is a measure of the equipment’s performance, indicating the probability of survival during a period of time, knowing that the equipment has not failed yet. This probability can be used to calculate the mean residual lifetime (MRL), and the optimal replacement policy for the equipment. If the probability is calculated while assuming that the equipment has not yet been put to work, it indicates the unconditional reliability of the equipment. When a condition monitoring system is available, analysts are interested in knowing the reliability based on the latest available information on the equipment’s degradation state, i.e. the conditional reliability, while taking into consideration the information obtained from the condition monitoring system. The MRL function is very important in reliability and survival analysis because it describes the aging process. More specifically, if the random variable *X* represents the life of a component, then MRL is given by

$$\begin{aligned} \mu (t)&= {} E[X-t|X>t] \\&= {} \frac{1}{\overline{G}\left( t\right) }\int \nolimits _{t}^{\infty } {\overline{G}}(x) dx,\quad t>0. \end{aligned}$$

Although the MRL function is defined for any random variable *X*, it is of particular interest when *X* is a non-negative random variable because it can then be thought of as a lifetime of a device, and then represents the conditional expected residual life of the device at time given that the device is still active at time *t*. In replacement and repair strategies, although the shape of the failure rate function plays an important role, the MRL function is found to be more relevant than the HR function because the former summarizes the entire residual life function, whereas the latter considers only the risk of instantaneous failure at some time. The MRL function of a random variable *X* with M–O log-logistic is

8 $$\mu (t) =\frac{t^{\beta }+\alpha \gamma ^{\beta }}{\alpha \gamma ^{\beta }}\int \nolimits _{t}^{\infty }\frac{\alpha \gamma ^{\beta }}{x^{\beta }+\alpha \gamma ^{\beta }}dx.$$

The value in Eq. (8) can be obtained numerically. Table 1 displays the mean residual life at point $t=2$ for M–O log-logistic at $\beta =3,\gamma =2$ and different choices of parameter $\alpha$.

**Table 1:** Mean residual life of M–O log-logistic

$\alpha$	$\beta$	$\gamma$	MRL at $t=2$
0.3	3	2	1.16808
0.7	3	2	1.36339
1.5	3	2	1.69029
2.5	3	2	2.02493

**Fig. 2:**
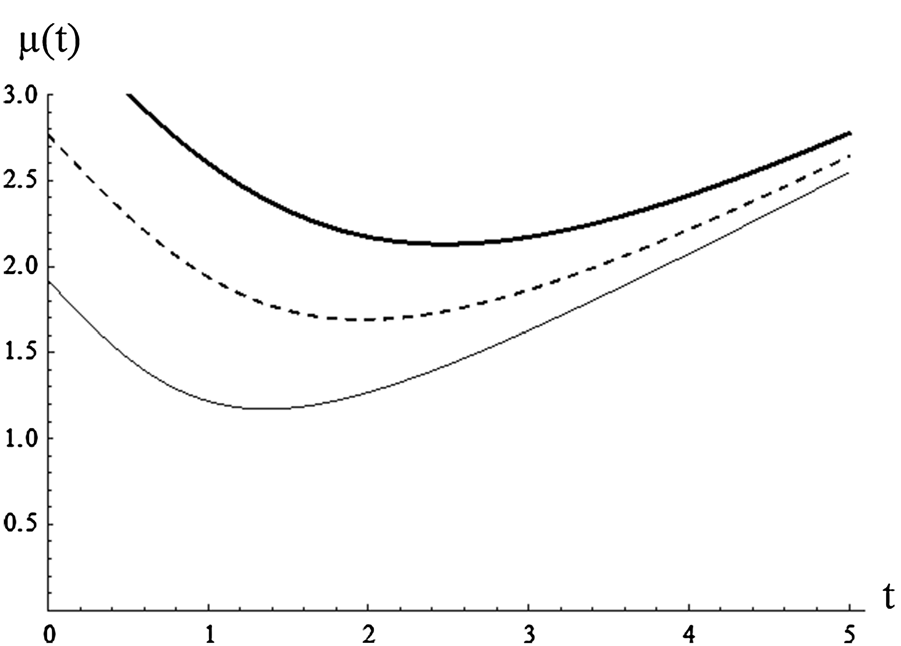
Plot of MRL for $\alpha =0.5,$ 1.5, 3 (*plain*, *dashed*, *bold*) , $\beta =3, \gamma =2$

From the above example, it is noted that the mean residual life is generally increasing for increasing values of $\alpha$ (Fig. 2).

Another interesting reliability function is the mean inactivity time (MIT) function (also known as the mean past lifetime and the mean waiting time functions). This function is well-known reliability measure which has several applications in many disciplines such as reliability theory, survival analysis, and actuarial studies. The MIT function of *X* is defined by

$$m(t) =\frac{1}{G(t)}\int \nolimits _{0}^{t}G(x)dx,\quad t>0.$$

The MIT function of a random variable *X* with M–O log-logistic is

9 $$m(t) =\frac{t^{\beta }+\alpha \gamma ^{\beta }}{t^{\beta }} \int \nolimits _{0}^{t}\frac{x^{\beta }}{x^{\beta }+\alpha \gamma ^{\beta }}dx.$$

The value of the function in (9) can be obtained by a numerical calculation. Table 2 displays the mean inactivity time at point $t=2$ for M–O log-logistic at $\beta =3, \gamma =2$ and different choices of parameter $\alpha$ (Additional file 2).

**Table 2:** Mean inactivity time of M–O log-logistic

$\alpha$	$\beta$	$\gamma$	MIT at $t=2$
0.3	3	2	0.84578
0.7	3	2	0.703955
1.5	3	2	0.614498
2.5	3	2	0.574372

**Fig. 3:**
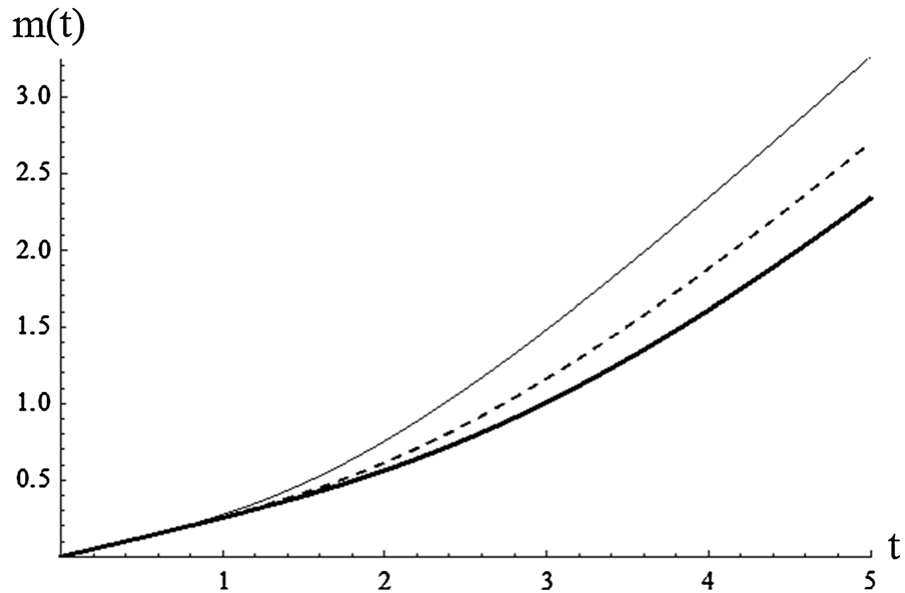
Plot of MIT for $\alpha =0.5$, 1.5, 3 (*plain*, *dashed*, *bold*) , $\beta =3$, $\gamma =2$)

From the above example, it is noted that the mean inactivity time is generally decreasing for increasing values of $\alpha$ (Fig. 3).

Recently, a reliability measure called strong mean inactivity time (SMIT) function has been introduced and studied (see Kayid and Izadkhah 2014). The SMIT function of a random variable *X* with M–O log-logistic is

10 $$\begin{aligned} M_{_{T}}(t)&= {} \frac{1}{G(t) }\int \nolimits _{0}^{t}2x G(x) dx \nonumber \\&= {} \frac{2\left[ t^{\beta }+\alpha \gamma ^{\beta }\right] }{t^{\beta }} \int \nolimits _{0}^{t}\frac{x^{\beta +1}}{x^{\beta }+\alpha \gamma ^{\beta }} dx. \end{aligned}$$

Table 3 displays the strong mean inactivity time at point $t=2$ for M–O log-logistic at $\beta =3,\gamma =2$ and different choices of parameter $\alpha$ (Additional file 3).

**Table 3:** Strong mean inactivity time of M–O log-logistic

$\alpha$	$\beta$	$\gamma$	SMIT at $t=2$
0.3	3	2	2.48358
0.7	3	2	2.14164
1.5	3	2	1.911302
2.5	3	2	1.804316

**Fig. 4:**
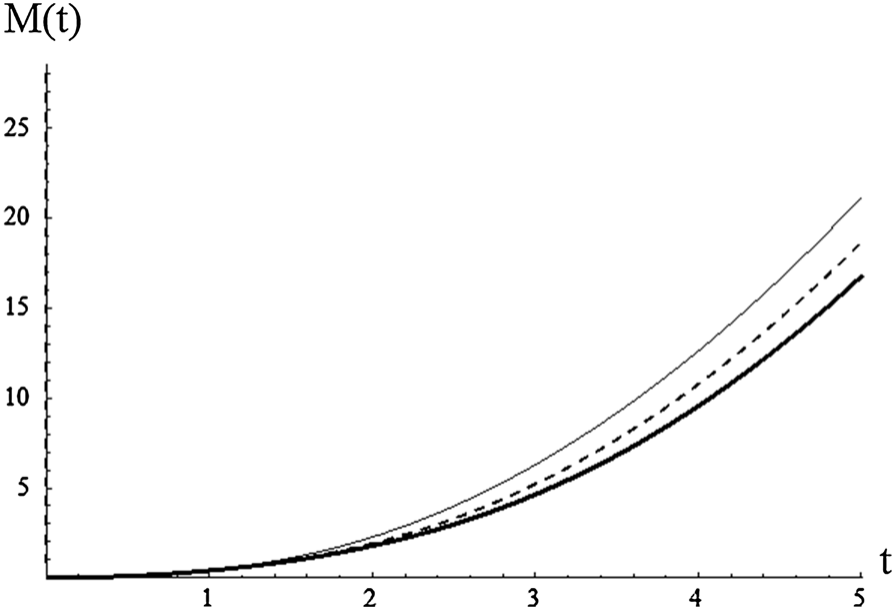
Plot of SMIT for ${\alpha =0.5,} {1.5,3}$ (*plain*, *dashed*, *bold*), ${\beta =3,\gamma =2}$

From the above example, it is noted that the strong mean inactivity time is generally decreasing for increasing values of $\alpha$ (Fig. 4).

### Mean, variance

We consider a random variable *X* with M–O log-logistic ($\alpha$, $\beta$, $\gamma$). The mean and variance are given, respectively, by

11 $$E(X) =\int \nolimits _{0}^{\infty }\frac{x\alpha \frac{\beta }{ \gamma }\left( \frac{x}{\gamma }\right) ^{\beta -1}}{\left( \left( \frac{x}{ \gamma }\right) ^{\beta }+\alpha \right) ^{2}}dx,$$

and

$$E(X^{2}) =\int \nolimits _{0}^{\infty }\frac{x^{2}\alpha \frac{\beta }{\gamma }\left( \frac{x}{\gamma }\right) ^{\beta -1}}{\left( \left( \frac{x}{\gamma }\right) ^{\beta }+\alpha \right) ^{2}}dx.$$

Hence

12 $$Var\left( X\right) =\int \nolimits _{0}^{\infty }\frac{x^{2}\alpha \frac{\beta }{\gamma }\left( \frac{x}{\gamma }\right) ^{\beta -1}}{\left( \left( \frac{x }{\gamma }\right) ^{\beta }+\alpha \right) ^{2}}dx-\left[ \int \nolimits _{0}^{\infty }\frac{x\alpha \frac{\beta }{\gamma }\left( \frac{x}{ \gamma }\right) ^{\beta -1}}{\left( \left( \frac{x}{\gamma }\right) ^{\beta }+\alpha \right) ^{2}}dx\right] ^{2}.$$

**Fig. 5:**
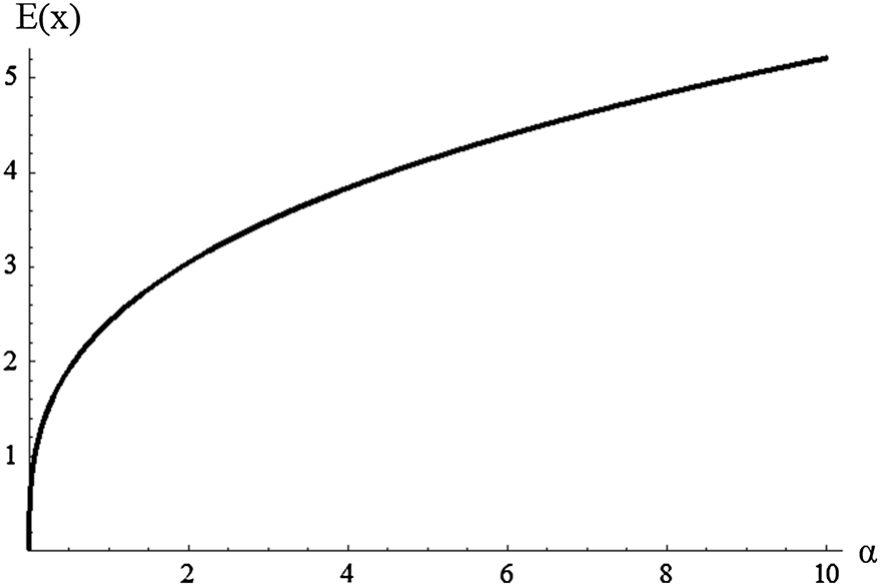
Plot of the mean of M–O log-logistic for different value of $\alpha$ and $\beta =3$, ${\gamma =2}$

**Fig. 6:**
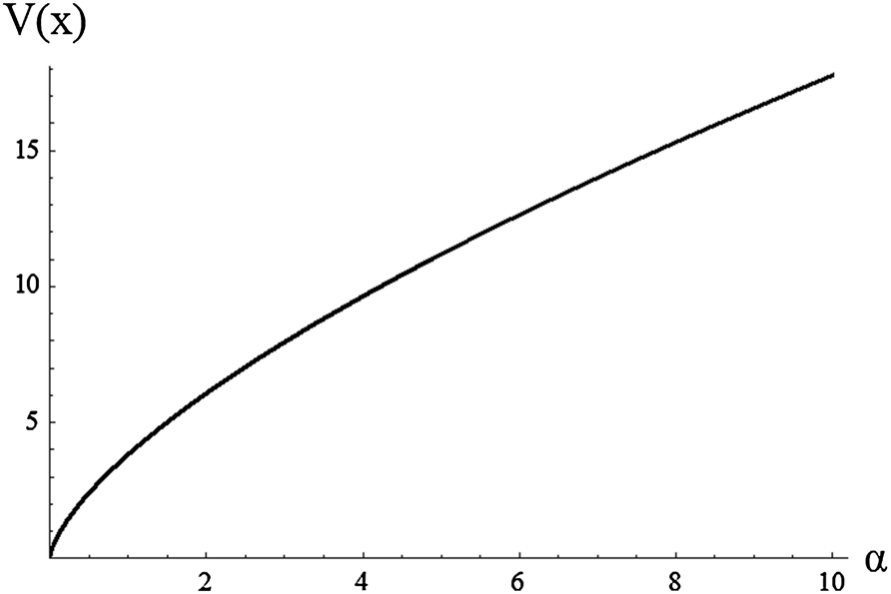
Plot of the variance of M–O log-logistic for different value of $\alpha$ and $\beta =3$, $\gamma =2$

In general, the last integrals cannot be given explicitly in terms of $\alpha ,\beta ,\gamma$. The mean E(X) and the variance Var(X) of M–O log-logistic are shown graphically in Figs. 5 and 6 for different value of $\alpha$ and $\beta =3$, $\gamma =2$. These figures show the mean and variance increase as the value of $\alpha$ increases.

### Median

Let *X* be a random variable with M–O log-logistic $(\alpha ,\beta ,\gamma )$. The median of this distribution is the value *m* that satisfies

13 $$\int \nolimits _{0}^{m}\frac{\alpha \frac{\beta }{\gamma }\left( \frac{x}{ \gamma }\right) ^{\beta -1}dx}{\left[ \left( \frac{x}{\gamma }\right) ^{\beta }+\alpha \right] ^{2}}=0.5$$

Table 4 displays the median for M–O log-logistic at $\beta =2$, $\gamma =0.2$ and different choices of parameter $\alpha$. It is noted that the median is generally increasing for increasing values of $\alpha$.

**Table 4:** Median of M–O log-logistic

$\alpha$	$\beta$	$\gamma$	Median
0.3	2	0.2	0.109545
0.7	2	0.2	0.167332
1.3	2	0.2	0.228035
2	2	0.2	0.282843

### Renyi entropy

Entropy has been used in various situations in science and engineering. The entropy of a random variable *X* with density function *g*(*x*) is a measure of variation of the uncertainty. The Renyi entropy of order $\delta$ is defined by

$$H_{\delta }(x)=\frac{1}{1-\delta }\log \left( \int \nolimits _{-\infty }^{\infty }g(x)^{\delta }dx\right) ,\quad \delta \ge 0,\quad \delta \ne 1.$$

Let *X* be a random variable with M–O log-logistic $(\alpha ,\beta ,\gamma )$. The corresponding Renyi entropy is obtained as

14 $$H_{\delta }(x)=\frac{1}{1-\delta }\log \left\{ \int \nolimits _{0}^{\infty }\left( \frac{\alpha \frac{\beta }{\gamma }\left( \frac{x}{\gamma }\right) ^{\beta -1}}{\left[ \left( \frac{x}{\gamma }\right) ^{\beta }+\alpha \right] ^{2}}\right) ^{\delta }dx\right\} ,\quad \delta \ge 0,\quad \delta \ne 1.$$

Table 5 displays the Renyi entropy for M–O log-logistic at $\delta =3, \beta =1.5,\gamma =1$ and different choices of the parameter $\alpha$. It is noted that the Renyi entropy is generally increasing for increasing values of $\alpha$.

**Table 5:** Renyi entropy of M–O log-logistic

$\alpha$	$\beta$	$\gamma$	Renyi entropy
0.3	1.5	1	0.12391
0.7	1.5	1	0.68877
1.5	1.5	1	1.19687
2	1.5	1	1.38866

## Maximum likelihood estimators

In statistics, maximum-likelihood estimation (MLE) is a method of estimating the parameters of a statistical model. When applied to a data set and given a statistical model, MLE provides estimates for the model’s parameters. The method of maximum likelihood corresponds to many well-known estimation methods in statistics.

Let $X_{1},\ldots ,X_{n}$ be a random sample from M–O log-logistic $(\alpha ,\beta ,\gamma )$, the likelihood function is given by

$$L\left( X_{1},\ldots ,X_{n}|\alpha ,\sigma ,\beta ,\gamma \right) =\frac{\alpha ^{n}\left( \frac{\beta }{\gamma }\right) ^{n}\left[ \prod \nolimits _{i=1}^{n}(\frac{x_{i}}{\gamma })^{\beta -1}\right] }{ \prod \nolimits _{i=1}^{n}\left[ \left( \frac{x_{i}}{\gamma }\right) ^{\beta }+\alpha \right] ^{2}}.$$

The logarithm of the likelihood function is then given by

15 $$\begin{aligned} \ell \left( X_{1},\ldots ,X_{n}|\alpha ,\sigma ,\beta ,\gamma \right)&= {} n\ln \alpha +n\ln \left( \frac{\beta }{\gamma }\right) +\left( \beta -1\right) \sum \limits _{i=1}^{n}\ln \left( \frac{x_{i}}{\gamma }\right) \nonumber \\&\quad -2\sum \limits _{i=1}^{n}\ln (\left( \frac{x_{i}}{\gamma }\right) ^{\beta }+\alpha ). \end{aligned}$$

The maximum likelihood estimators (MLEs) of $\alpha ,\beta$ and $\gamma$ can be obtained by solving the nonlinear equations

16 $$\frac{\partial \ell }{\partial \alpha }= {} \frac{n}{\alpha }-2\sum \limits _{i=1}^{n}\frac{1}{\left( \frac{x_{i}}{\gamma }\right) ^{\beta }+\alpha }=0$$

17 $$\begin{aligned} \frac{\partial \ell }{\partial \beta }&= {} \frac{n}{\beta }+\sum \limits _{i=1}^{n}\ln \left( \frac{x_{i}}{\gamma }\right) -2\sum \limits _{i=1}^{n}\frac{\left( \frac{x_{i}}{\gamma }\right) ^{\beta }\ln \left( \frac{x_{i}}{\gamma }\right) }{\left( \frac{x_{i}}{\gamma } \right) ^{\beta }+\alpha }=0 \end{aligned}$$

18 $$\frac{\partial \ell }{\partial \gamma }= -\frac{n}{\gamma }-\frac{n\left( \beta -1\right) }{\gamma }+2\sum \limits _{i=1}^{n}\frac{\beta x_{i}^{\beta }}{ \left( \left( \frac{x_{i}}{\gamma }\right) ^{\beta }+\alpha \right) \gamma ^{\beta +1}}=0$$

There is no explicit solution for Eqs. (16)–(18), so they need to be solved numerically. For a given known scale parameter $(\gamma =1),$ 1000 different samples are simulated from M–O log-logistic with different sizes and different values of the scale parameter $\alpha$. We studied the behavior of the MLEs from unknown scale parameter $\alpha$ and shape parameter $\beta$. The values of $\alpha$ are taken as 0.8, 1.5, and 2.5, while the value of $\beta$ is 2. Tables 6 and 7 represent MLEs of parameter $\alpha$ and $\beta$, respectively.

**Table 6:** MLE of the parameter ${\small \alpha }$

${\alpha }$	*n*	Estimate	Bias	MSE
0.3	20	0.296275	−0.00372454	0.0237631
	50	0.231197	−0.0688027	0.0182828
	70	0.252118	−0.047882	0.0128916
	150	0.285643	−0.0143573	0.00492491
1.2	20	1.02572	−0.174276	0.291608
	50	1.20359	0.00358953	0.103632
	70	1.12847	−0.0715284	0.119474
	150	1.19823	−0.00177408	0.0339848
2.5	20	2.34865	−0.151351	1.2994
	50	2.59336	0.0933644	0.517638
	70	2.57025	0.0702524	0.392246
	150	2.53202	0.0320212	0.151326

**Table 7:** MLE of the parameter ${\beta }$

$\beta$	*n*	Estimate	Bias	MSE
${\alpha =0.3}$	20	1.91064	−0.0893629	0.394056
	50	2.02933	0.0293271	0.0744106
	70	1.9975	−0.00249889	0.0850081
	150	2.01348	0.0134826	0.0190133
${\alpha =1.2}$	20	1.96364	−0.036358	0.341544
	50	2.03782	0.0378182	0.0577503
	70	2.02835	0.0283514	0.0409679
	150	2.01348	0.0134826	0.0190133
${\alpha =2.5}$	20	1.94389	−0.0561107	0.372105
	50	1.9975	−0.00249889	0.0850081
	70	2.02563	0.0283514	0.0409679
	150	2.01095	0.0109501	0.0227298

From Table 6 it is observed that the estimate, mean square error and the bias of the MLE of the parameter $\alpha$ are decreasing when the sample size(*n*) is increasing. From Table 7 it is observed that the estimate, mean square errors, and the bias of the MLE of the parameter $\beta$ are decreasing when the sample size (*n*) is increasing. The second derivatives of (16)–(18) are

$$\begin{aligned} \frac{\partial ^{2}\ell }{\partial \alpha ^{2}}&= {} \frac{-n}{\alpha ^{2}} +2\sum \limits _{i=1}^{n}\frac{1}{\left( \left( \frac{x_{i}}{\gamma }\right) ^{\beta }+\alpha \right) ^{2}}.\\ \frac{\partial ^{2}\ell }{\partial \beta ^{2}}&= {} \frac{-n}{\beta ^{2}} -2\sum \limits _{i=1}^{n}\frac{\left( \frac{x_{i}}{\gamma }\right) ^{\beta }\left( \ln \left( \frac{x_{i}}{\gamma }\right) \right) ^{2}\left( \left( \frac{x_{i}}{\gamma }\right) ^{\beta }+\alpha \right) -\left( \left( \frac{ x_{i}}{\gamma }\right) ^{\beta }\ln \left( \frac{x_{i}}{\gamma }\right) \right) ^{2}}{\left( \left( \frac{x_{i}}{\gamma }\right) ^{\beta }+\alpha \right) ^{2}}.\\ \frac{\partial ^{2}\ell }{\partial \gamma ^{2}}&= {} \frac{n}{\gamma ^{2}} +\frac{n\left( \beta -1\right) }{\gamma ^{2}}+2\sum \limits _{i=1}^{n}\frac{ \beta x_{i}^{\beta }\left[ \beta x_{i}^{\beta }-\left( \beta +1\right) \gamma ^{\beta }\left( \left( \frac{x_{i}}{\gamma }\right) ^{\beta }+\alpha \right) \right] }{\left( \left( \frac{x_{i}}{\gamma }\right) ^{\beta }+\alpha \right) ^{2}\gamma ^{2\left( \beta +1\right) }}\\ \frac{\partial ^{2}\ell }{\partial \alpha \partial \beta }&= {} 2\sum \limits _{i=1}^{n}\frac{\left( \frac{x_{i}}{\gamma }\right) ^{\beta }\ln \left( \frac{x_{i}}{\gamma }\right) }{\left( \left( \frac{x_{i}}{\gamma }\right) ^{\beta }+\alpha \right) ^{2}}.\\ \frac{\partial ^{2}\ell }{\partial \alpha \partial \gamma }&= {} -2\sum \limits _{i=1}^{n}\frac{\beta x_{i}^{\beta }}{\left( \left( \frac{x_{i} }{\gamma }\right) ^{\beta }+\alpha \right) ^{2}\gamma ^{\beta +1}}.\\ \frac{\partial ^{2}\ell }{\partial \beta \partial \gamma }&= {} - \frac{n}{\gamma }+2\sum \limits _{i=1}^{n}\frac{\left( \left( \frac{x_{i}}{ \gamma }\right) ^{\beta }+\alpha \right) \left( \frac{\beta x_{i}^{\beta }\ln \left( \frac{x_{i}}{\gamma }\right) +x_{i}^{\beta }}{\gamma ^{\beta +1}} \right) -\frac{\beta x_{i}^{2\beta }\ln \left( \frac{x_{i}}{\gamma }\right) }{\gamma ^{2\beta +1}}}{\left( \left( \frac{x_{i}}{\gamma }\right) ^{\beta }+\alpha \right) ^{2}}. \end{aligned}$$

If we denote the MLE of $\theta =(\alpha ,\beta ,\gamma )$ by $\hat{\theta } =( \hat{\alpha },\hat{\beta },\hat{\gamma })$, the observed information matrix is then given by

$$\begin{aligned} I(\theta ) = \left[ \begin{array}{ccc} -\frac{\partial ^{2}\ell }{\partial \alpha ^{2}} &{} -\frac{\partial ^{2}\ell }{\partial \alpha \partial \beta } &{} -\frac{\partial ^{2}\ell }{ \partial \alpha \partial \gamma } \\ -\frac{\partial ^{2}\ell }{\partial \alpha \partial \beta } &{} -\frac{ \partial ^{2}\ell }{\partial \beta ^{2}} &{} -\frac{\partial ^{2}\ell }{ \partial \beta \partial \gamma } \\ -\frac{\partial ^{2}\ell }{\partial \alpha \partial \gamma } &{} -\frac{ \partial ^{2}\ell }{\partial \beta \partial \gamma } &{} -\frac{\partial ^{2}\ell }{\partial \gamma ^{2}} \end{array}\right] \end{aligned}$$

Hence the variance covariance matrix would be $I^{-1}( \theta )$. The approximate $(1-\delta )100\,\%$ confidence intervals (CIs) for the parameters $\alpha$, $\beta$ and $\gamma$ are $\hat{\alpha }\pm Z_{\frac{ \delta }{2}}V(\hat{\alpha })$, $\hat{\beta }\pm Z_{\frac{\delta }{ 2}}V(\hat{\beta })$ and $\hat{\gamma }\pm Z_{\frac{\delta }{2} }V( \hat{\gamma })$ respectively, where $V(\hat{\alpha } )$, $V(\hat{\beta })$ and $V( \hat{\gamma })$ are the variances of $\hat{\alpha }$, $\hat{\beta }$ and $\hat{\gamma }$, which are given by the diagonal elements of $I^{-1}(\theta )$, and $Z_{\frac{\delta }{2}}$ is the upper $(\delta {/}2)$ percentile of standard normal distribution.

## Fitting reliability data

In this section, we provide two data sets analysis to show how the model works in practice.

### First data set

The first data set given in Gupta et al. (1999) is about days of survival for lung cancer patients

**Table:** Data set

389	18	22	10	112	63	100	13	151	467
162	117	122	33	42	99	283	80	314	112

Some properties of the data set were computed in Table 8.

**Table 8:** Some properties of data set

*E*(*X*)	*Var*(*X*)	Kurtosis	Skewness
135.45	16,735.1	0.526142	1.26934

From the above table, it is clear that the distribution of this data set is positively skewed right and leptokurtic. The parameter of the sample is estimated numerically. We used Eqs. (16)–(18) to obtain MLEs estimate and the results are given in Table 9.

**Table 9:** MLE for data set

Parameter	MLE
${\alpha }$	10.833
${\beta }$	1.58621
${\gamma }$	19.7451

If we want to test if this data fits the M–O log-logistic $(\alpha ,\beta ,\gamma )$, our hypotheses is $H_{0}:F$ = *F*_M–O log-logistic_ versus $H_{1}:F\ne$  *F*_M–O log-logistic_. We use the Kolmogorov–Smirnov (*K*–*S*) distances between the empirical distribution function and the fitted distribution function to determine the appropriateness of the model. *K*–*S* at 95 % CIs value and the corresponding *p* value are presented in Table 10.

**Table 10:** The *K*–*S* and *p* value of data set

*K*–*S*	*p* value
0.103124	0.131

The small K–S distance and the large *p* value for the test indicate this data fits the M–O log-logistic quite well. Also we use likelihood ratio test (LRT) to determine the appropriateness of the model. The hypotheses are as follows:

$$H_{0}:\alpha =1(log{\hbox {-}}logistic)\quad \text {versus}\quad H_{1}:\alpha \ne 1(M{{-}}O\,log{\hbox {-}}logistic).$$

The *log-likelihood* value, likelihood ratio statistic $( \Lambda )$ and corresponding *p* value are presented in Table 11.

**Table 11:** The result of likelihood ratio test

*Log-likelihood*	$\Lambda$	*p* value
$-118.851$	32.7814	1.03127 × 10^−8^

We note that the calculated LRT statistic is greater than the critical point for this test, which is 6.635, and also that the *p* value is very small. According to the LRT, we conclude that this data fits the M–O log-logistic much better than the log-logistic distribution.

### Second data set

The second data set obtained from www.isixigma.com represents a cycle time of a process.

**Table:** Data set

10	13	13	14	14	15	15	16	25	26	26	27	38	53
17	17	17	17	18	18	18	19	27	27	28	28	42	
21	21	21	22	22	23	24	25	30	34	35	35	42	

Some properties of the data set were computed in Table 12.

**Table 12:** Some properties of data set

*E*(*X*)	*Var*(*X*)	Kurtosis	Skewness
23.825	86.1481	0.934659	1.05974

From the above table, it’s clear that the distribution of this data set is positively skewed right and leptokurtic. The parameter of the sample is estimated numerically. We used Eqs. (16)–(18) to obtain MLEs estimate and the results are given in Table 13.

**Table 13:** MLE for data set

Parameter	MLE
${\alpha }$	0.457038
${\beta }$	4.70536
${\gamma }$	26.0741

We want to test if these data fit the M–O log-logistic or not, our hypotheses is $H_{0}:F$ = *F*_M–O log-logistic_ versus $H_{1}:F\ne$  *F*_M–O log-logistic_. We use the *K*–*S* distances between the empirical distribution function and the fitted distribution function to determine the appropriateness of the model. *K*–*S* value at 99 % CIs and the corresponding *p* value are presented in Table 14.

**Table 14:** The *K*–*S* and *p* value of data set

*K*–*S*	*p* value
0.0982563	0.212

The small K–S distance and the large p-value for the test indicate that this data fits the M–O log-logistic quite well. Also we use LRT to determine the appropriateness of the model. The hypotheses are as follow:

$$H_{0}:\alpha =1(log{\hbox {-}}logistic)\quad \text {versus}\quad H_{1}:\alpha \ne 1(MO\,log{\hbox {-}}logistic).$$

Log-likelihood value, likelihood ratio statistic $(\Lambda )$ and corresponding *p* value are presented in Table 15.

**Table 15:** The result of likelihood ratio test

*Log-likelihood*	$\Lambda$	*p* value
−141.405	7.84139	0.00510632

We note that the calculated LRT statistic is greater than the critical point for this test, which is 6.635, and also that the *p* value is very small. According to the LRT, we conclude this data fits the M–O log-logistic much better than the log-logistic distribution.

## Conclusion

In this paper, an extended model based on log-logistic distribution is investigated. Some reliability and statistical properties of this model are obtained. Through numerical simulation, the MLE of the parameters are calculated and discussed. Finally, two sets of real data are fitted to this model and is shown to be appropriate. Further properties and applications of the model can be considered in the future of this research. In particular, the following topics are interesting and still remain as open problems:

1. Discuss the Bayesian analysis of the model.
2. Introduce and study a new class of weighted M–O bivariate log-logistic distribution.

## Additional files

10.1186/s40064-016-2007-x First data set.
10.1186/s40064-016-2007-x Second data set.
10.1186/s40064-016-2007-x Simulation results.
